# The many faces of the GID/CTLH E3 ligase complex

**DOI:** 10.1042/BST20253074

**Published:** 2025-10-22

**Authors:** Arno F. Alpi, Jakub Chrustowicz, Dawafuti Sherpa, Brenda A. Schulman

**Affiliations:** 1 Department of Molecular Machines and Signaling, Max Planck Institute of Biochemistry, Martinsried, 82152, Germany; 2 Institute of Cell Biology, School of Biological Sciences, University of Edinburgh, Edinburgh, U.K.

**Keywords:** degron, E3 ubiquitin ligase, GID/CTLH E3, higher-order E3 assembly, molecular glue, oligomeric metabolic enzymes, ubiquitin proteasome system

## Abstract

The GID/C-terminal to LisH (CTLH) E3 is an emerging family of evolutionarily conserved multiprotein E3 ligase complexes implicated in various biological processes including metabolic rewiring, stress-responsive regulation, cellular differentiation, and immunity. Pioneering biochemical reconstitution, cryo-EM, and cell-based studies have illuminated many aspects of the compositional and structural dynamics of GID/CTLH E3 complexes. GID/CTLH E3 undergoes sophisticated regulation through incorporation of interchangeable substrate receptors and association with supramolecular assembly factors enabling higher-order complex formation. Furthermore, paralogous subunits vary and may modulate function across cell types. Additionally, an assortment of regulatory factors fine-tune substrate selection, underscoring the adaptability of this E3 ligase system. Here, we review these distinct ubiquitin ligase features, examine the mechanistic implications of GID/CTLH E3 regulation and the exquisite targeting of oligomeric substrates, and discuss potential for therapeutic application in targeted protein degradation.

## Introduction to GID/CTLH E3 ligases

Specificity of the ubiquitin system is determined by E3 ubiquitin ligases, which recruit specific substrates for modification [1]. For the majority of E3s, catalysis of ubiquitylation depends on one or more RING or RING-like domains. RING/RING-like domains typically bind a ubiquitin-loaded E2 conjugating enzyme and promote transfer of ubiquitin’s C-terminus from a reactive thioester linkage with the E2’s catalytic cysteine to a lysine on the E3-bound substrate [2,3]. This mechanism is conserved for a family of multiprotein E3 complexes termed GID in yeast and C-terminal to LisH (CTLH) in humans, and their cognate partner E2s Ubc8 and UBE2H, respectively. However, recent studies have revealed that the GID/CTLH E3 ligases also undergo a remarkable series of distinct structural transformations. Incorporation of interchangeable and variable subunits and formation of higher-order assemblies is required for regulation and substrate specificity.

**Figure 1:**
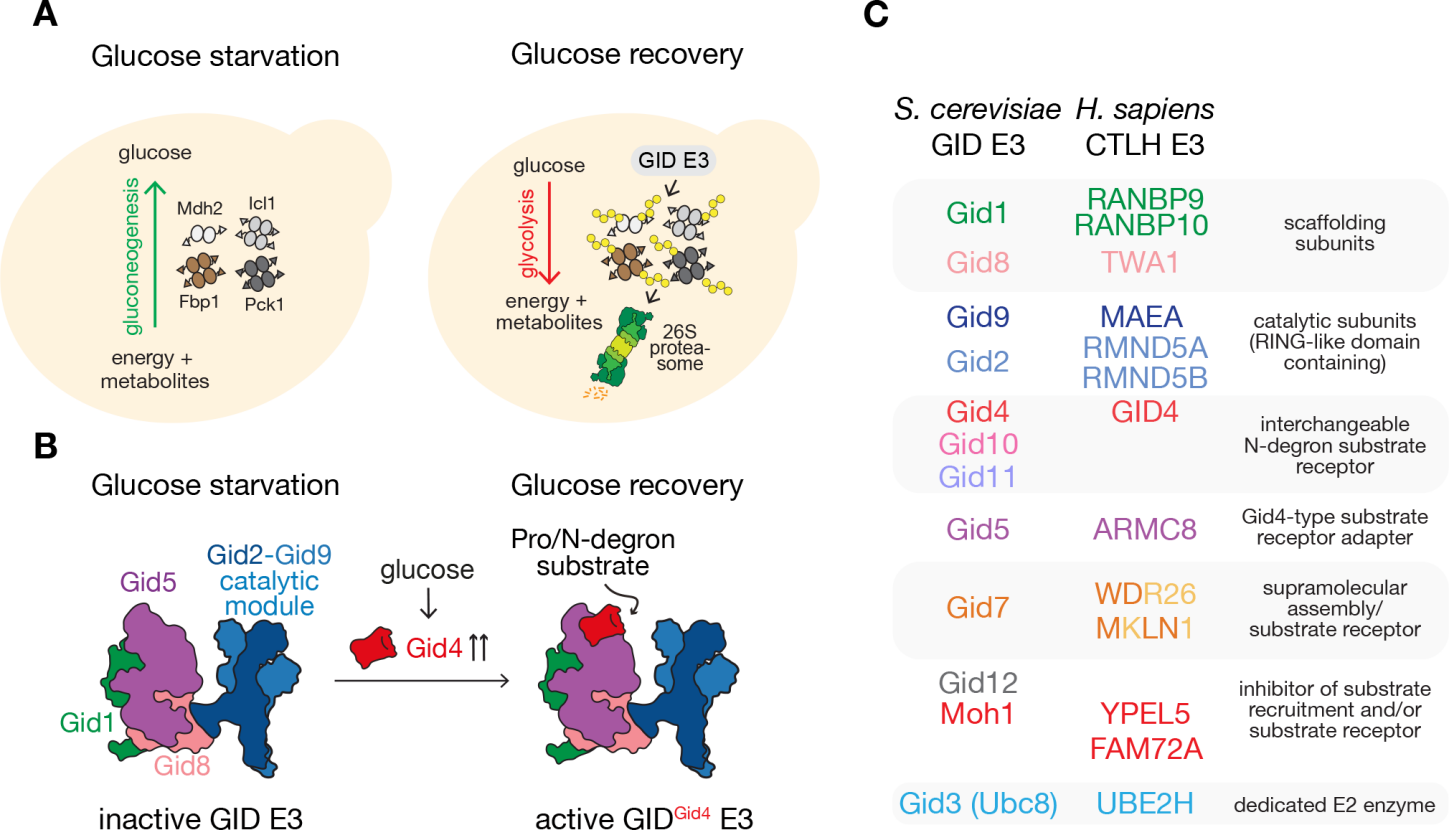
The yeast GID E3 ligase modulates glucose metabolism. (**A**) Upon glucose starvation, yeast cells carry out gluconeogenesis to produce glucose (left). Glucose recovery triggers GID E3 ligase-dependent ubiquitylation (yellow circles) of key gluconeogenic enzymes (Mdh2, Fbp1, Icl1, Pck1) and their degradation by the 26S proteasome, thereby promoting adaptation to glycolytic conditions (right). (**B**) Structure-based cartoon representation of the inactive GID E3 complex assembly under glucose starvation condition. The substrate-receptor adaptor subunit Gid5 (violet), scaffolding subunits Gid1 (green) and Gid8 (salmon), and the catalytic module (blue) are indicated. In response to glucose recovery, the substrate receptor Gid4 is expressed to assemble an active GID^Gid4^ E3 complex. Through its binding to Gid5, the Gid4 substrate-binding pocket is oriented toward the catalytic module to facilitate Pro/N-degron substrate binding and ubiquitylation. (**C**) Table listing conserved subunits of the *S. cerevisiae* GID E3 and the *H. sapiens* CTLH E3 complex grouped by their common functions.

The GID E3 complex was originally discovered in *S. cerevisiae* for co-ordinating the reciprocally regulated pathways of glycolysis and gluconeogenesis [4]. Yeast cells, when cultured on non-fermentable carbon sources such as ethanol, require gluconeogenesis [5]. Gluconeogenesis produces glucose through an enzymatic cascade involving Fructose-1,6-bisphosphatase (Fbp1, rate-limiting enzyme in the pathway), Phosphoenolpyruvate carboxykinase (Pck1), Malate dehydrogenase (Mdh2), and Isocitrate lyase (Icl1) [5]. When gluconeogenesis becomes dispensable through switching yeast from carbon starvation to glucose-rich media, Fbp1 and other key gluconeogenic enzymes are eliminated by ubiquitin-mediated degradation (Figure 1A) [5–7]. The regulatory gene products were identified in screens led by the Wolf group [8], who discovered that Gid1, Gid2, Gid4, Gid5, Gid7, Gid8, and Gid9 are subunits of a multiprotein E3 ligase complex termed GID (their mutation rendered yeast ‘*G*lucose-*i*nduced degradation-*d*eficient’) [4,9]. The subunits containing RING-like domains, Gid2 and Gid9, form a heterodimeric unit that catalyzes ubiquitylation partnering with the E2 enzyme Ubc8, also identified as Gid3 [4,8,10].

The question of how the GID E3 targets gluconeogenic enzymes was partially answered by the discovery that Gid4 is a substrate receptor (SR) [11]. Yeast regulate Gid4 expression and stability in response to fluctuating glucose levels [4,9]. Replenishment of glucose to carbon-starved yeast triggers Gid4 expression. Meanwhile, prolonged yeast cultivation in glucose-rich media leads to GID E3-dependent degradation of Gid4 [12]. Gid4 binds the Gid5 subunit within the GID complex, generating an active ‘GID^Gid4^’ E3 (Figure 1B) [4,9,12,13]. Gid4 recognizes the so-called ‘Pro/N-degrons’, distinguished by prolines at N-termini [11,14]. Pro/N-degrons are directly generated upon co-translational removal of the initiator methionine from Fbp1, Icl1, Mdh2, and through processing of Pck1 by Icp55 [11,14,15]. These initial studies in yeast established a GID E3 complex as a multiprotein, RING E3 ubiquitin ligase, regulated by metabolically controlled expression of a Pro/N-degron-binding substrate receptor.

The GID E3 complex subunits are evolutionarily conserved from yeast to humans, although known with differing subunit nomenclature (Figure 1C) [16–18]. Several key concepts emerged from early studies of the GID E3 subunits and complexes from higher organisms. First, while most GID E3 subunits display individualistic domain features, many share a domain termed ‘C-terminal to LisH (CTLH)’ which led to the name ‘CTLH’ for the GID E3 complexes in higher organisms [19]. It is now known that the CTLH domain forms a unit together with the upstream Lissencephaly-1 Homology (LisH) and the downstream CT11-RanBP9 (CRA) domains, promoting homotypic and heterotypic protein-protein interactions between subunits in GID/CTLH E3 complexes [13,20–22]. Also, there is not a strict 1:1 correspondence between GID/CTLH subunits across organisms, which reflect variations in assemblies and substrate regulation (Figure 1C). Finally, the role of a GID E3 complex in catabolite inactivation appears specialized for *S. cerevisiae*. CTLH E3s of higher eukaryotes have not yet been associated with the regulation of gluconeogenesis, but might be implicated in ubiquitin targeting of glycolytic enzymes associated with non-proteolytic regulation [23]. However, CTLH subunits have been associated with numerous crucial biological processes [18,24]. Development of flies and frogs requires RMND5, a CTLH E3 catalytic subunit [25–27], and mice deficient for the other catalytic subunit, MAEA, show abnormal hematopoiesis and are anemic [28,29]. Mammalian WDR26 (yeast Gid7 ortholog) has been associated with cellular signaling, cell proliferation, and nuclear condensation in erythropoiesis [30–36], whereas MKLN1 (a second yeast Gid7 ortholog) regulates cell morphology, immunity, and neuronal function [37–39]. Moreover, recent studies identified WDR26 and WDR26-associated subunit YPEL5 to play a role in host anti-microbial response and pyrimidine biosynthesis [40–42]. Thus, GID/CTLH E3 complexes clearly have functions beyond regulation of gluconeogenic enzymes.

Below, we describe structural and mechanistic studies that have revealed a suite of GID/CTLH E3 ligase complexes, varying by subunit composition, higher-order assembly, and activity.

## Regulation through N-degron recognition by yeast and human Gid4

**Figure 2:**
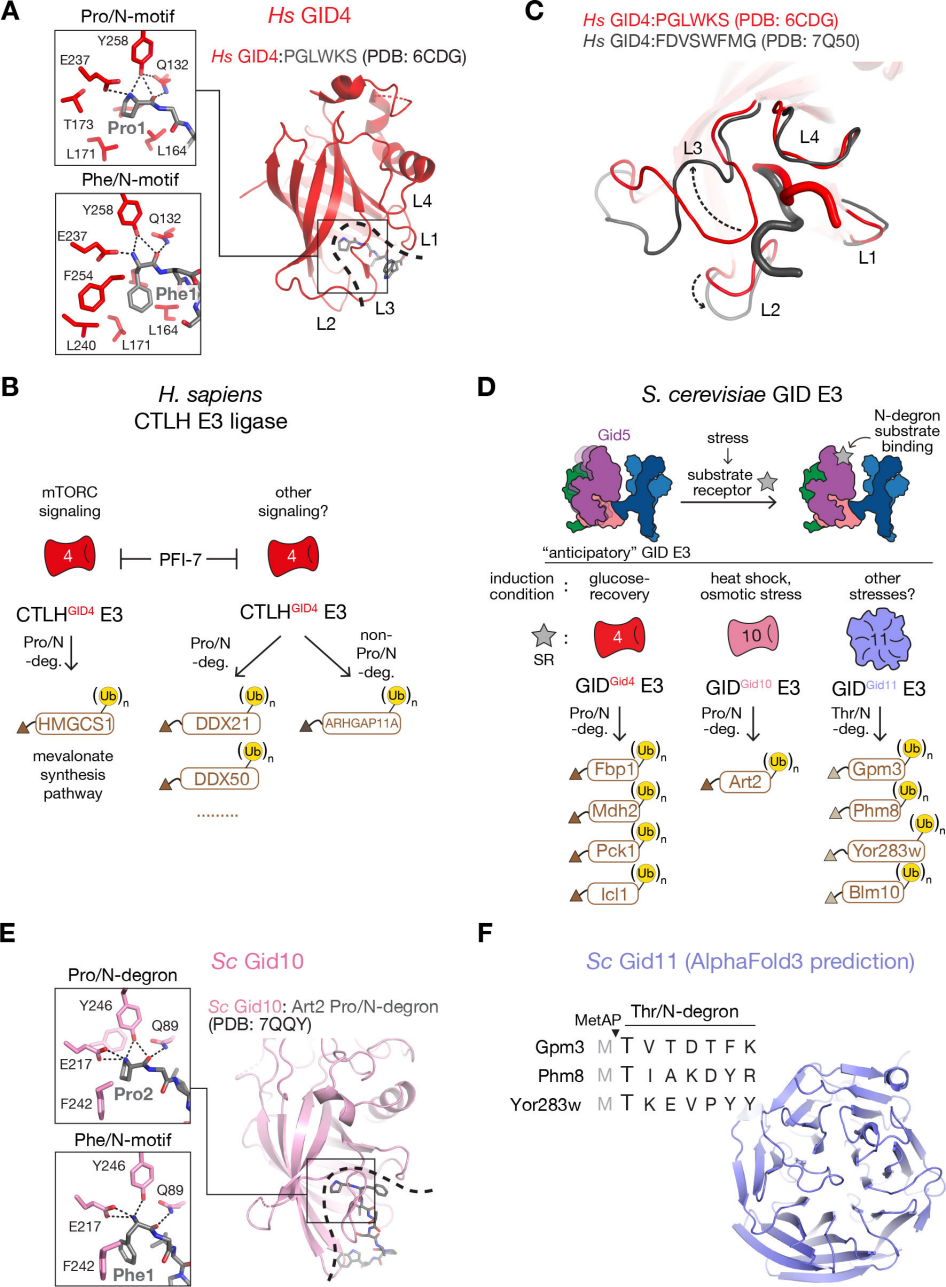
Regulation of the GID/CTLH E3 complex by interchangeable substrate receptors. (**A**) Crystal structure showing human GID4 substrate-binding groove (dashed line) bound to a Pro/N-motif (PGLWKS) (PDB: 6CDG) [46]. Close-ups highlight the residues at the bottom of GID4’s pocket anchoring the N-terminal amino groups of Pro (top) and non-Pro (bottom, PDB: 7Q50) [47]-initiating peptides and the analogous interactions contributing to recognition of their N-terminal residues. (**B**) The chemical probe PFI-7 antagonizing N-degron binding to GID4 was utilized to identify GID4-dependent interactors and potential Pro/N-degron and non-Pro/N-degron CTLH^GID4^ E3 substrates (brown triangles, N-degrons; (Ub)_n_, polyubiquitin chains). (**C**) Overlay of GID4 crystal structures highlighting conformational plasticity of its substrate-binding domain. Rearrangement of the β-hairpin loops L2 and L3 (indicated by black arrows) at the entrance to the GID4 pocket enables engagement of divergent Pro/ (PGLWKS, (PDB: 6CDG) [46] and non-Pro/N-motifs (FDVSWFMG, PDB: 7Q50) [47]. (**D**) Overview of activation of the pre-assembled ‘anticipatory’ GID E3 by the cellular stress-induced interchangeable substrate receptors Gid4, Gid10, Gid11, along with their corresponding N-degron substrates (brown triangles, N-degron; (Ub)_n_, polyubiquitin chains). (**E**) Crystal structure of yeast Gid10 bound to Art2 Pro/N-degron (PDB: 7QQY) [48]. Close-ups highlight the Gid10’s residues at the bottom of its substrate-binding domain (dashed line) mediating specificity for Art2’s N-terminal Pro2 (exposed upon co-translational processing by MetAP) and the non-Pro Phe/N-motif (FWLPANLW, PDB: 7Q51) [47]. (**F**) AlphaFold3 [49] prediction of the putative substrate-binding domain of yeast Gid11, along with sequence alignment of Gid11-dependent substrates’ N-termini, revealing their Thr/N-degron motifs (exposed upon removal of initiator Met).

Structural studies revealed how conserved Gid4-type subunits both bind substrates and associate into active E3 ligase complexes. Although human substrates recognized based on their N-terminal Pro were only recently discovered [43–45], model peptides were amenable to crystallization with the substrate-binding domain of human GID4 (Figure 2A) [46]. Importantly, a high-resolution cryo-EM structure showed yeast Gid4 within a fully-assembled GID E3 ligase binding a bona-fide Pro/N-degron, from Fbp1, through homologous interactions [20]. GID4’s peptide binding domain adopts a calycin-type β-barrel, with a funnel-shaped substrate binding tunnel that anchors the N-degron peptide [46]. Acidic and polar GID4 residues engage the amino group of an N-terminal Pro. Moreover, the specificity for the N-terminal Pro is dictated by a small hydrophobic GID4 pocket that accommodates the aliphatic Pro side chain. Also, the opening of the funnel-shaped GID4 comprises flexible loops that capture several residues downstream of an N-terminal Pro. This rationalizes why substrate selectivity is predominantly determined by the four N-terminal residues of the degron motif.

Recently, the development of PFI-7, a potent and selective chemical probe antagonizing N-degron binding to GID4, provided a powerful tool to identify GID4-dependent interactors and potential CTLH E3 candidate substrates (Figure 2B) [43,44]. Using PFI-7 in comparative quantitative proteomics studies revealed several potential substrates. Many proteins stood out for connections to RNA function, such as the Pro/N-motif-containing RNA-binding proteins DDX50 and DDX21 (Figure 2B) [43]. Moreover, mTORC1-regulated turnover of HMGCS1, an enzyme of the mevalonate synthesis pathway, depends on CTLH E3 activity [43,45,50]. The data suggest that GID4 recognizes the Pro/N-motif of HMGCS1 and promotes degradation upon mTORC1 inhibition by Torin-1[45]. Accordingly, PFI-7 inhibits Torin-1 induced HMGCS1 targeting [45,50]. Whether and how mTORC1 signaling regulates CTLH E3 activity is a compelling question for further investigation.

Several recent studies suggest potential for Gid4/GID4 to recognize substrates in addition to those with Pro/N-degrons. Systematic screens revealed both yeast Gid4 and human GID4 can bind peptides harboring non-Pro N-terminal residues Phe, Leu, Val, Ile, and Trp in the context of distinct downstream sequence motifs [47,51]. Structures showed that the ability of human GID4 to recognize diverse N-terminal sequences stems from the conformational pliability of its β-hairpin loops located at the rim of the β-barrel entrance (Figure 2C) [47,51]. The side chains of hydrophobic N-terminal residues dock into the same apolar GID4 pocket as an N-terminal Pro (Figure 2A and C). Interestingly, GID4’s groove rearranges to accommodate various sequences beyond the N-terminus [47,51].

The recently characterized CTLH E3 substrate ARHGAP11A, a member of the Rho-GAP family, provided evidence of a functional non-Pro/N-degron pathway regulated by human GID4[50]. ARHGAP11A harbors an N-terminal motif Trp-Asp-Gln-Arg, which matches a peptide sequence identified as binding GID4 by phage display [47]. Depletion of GID4 or GID4 inactivation by PFI-7 leads to accumulation of ARHGAP11A at the cell periphery where it inactivates RhoA and impairs cell motility [52].

## Regulation by interchangeable substrate receptors in yeast

The *S. cerevisiae* genome encodes two structurally homologous calycin-type β-barrel proteins, Gid4 and Gid10, which share high sequence similarity, bind the GID E3 complex via Gid5, and biochemically function as interchangeable substrate receptors [47,53,54]. One hint that these may differentially mediate regulation by the GID E3 is that Gid4 and Gid10 are expressed under different environmental conditions and stresses [48,53], suggesting non-redundant biological roles (Figure 2D). Gid10 is not induced by glucose but rather is up-regulated upon heat, starvation, and osmotic stresses [48,53]. Available data point to Gid10 serving as a Pro/N-degron substrate receptor for Art2 [48,55], a member of the α-arrestin family and best known as adaptor protein of the Rsp5 E3 ligase regulating plasma membrane nutrition transporters [56,57]. Crystal structures of Gid10 bound to Art2’s Pro/N-degron showed degron recognition analogous to that employed by both yeast and human Gid4, with specificity established by subtle differences in residues surrounding the central substrate-binding cavity (Figure 2E) [48,54]. Finally, consistent with the Gid10 pocket comprising pliable loops interacting with downstream degron residues, a peptide library screen revealed the capacity of Gid10 to recognize a distinct consensus motif GWLPPNLX enabled by structural plasticity of the loops [47].

In addition, a systems biology study uncovered a third yeast SR, Gid11, which associates with the GID E3 complex in a Gid5-dependent manner [58,59]. Unlike Gid4 and Gid10, recent studies suggest that Gid11 recognizes substrates with an N-terminal Thr residue (after Met cleavage) via a negatively charged pocket in the center of its structurally predicted WD40-repeat β-propeller domain (Figure 2F) [58–61]. β-propellers are common protein-interaction domains in various E3 ligase SRs [62]. Interestingly, Gid11 is expressed during the switch of carbon source from glucose to ethanol and assembles into the GID^Gid11^ E3 complex to target glycolysis-associated factors [59]. GID^Gid11^ E3 opposes the role of GID^Gid4^ E3 in targeting gluconeogenic enzymes during the reverse switch from ethanol to glucose, thus placing GID E3 complexes with interchangeable SRs as central regulators of metabolic transitions.

Yeast in particular seems to exploit the regulatory concept of SRs interchangeably binding to the same site on the GID E3, while only one Gid4-like SR (GID4) has been identified in higher eukaryotes [11,13,20,48,53,58]. Instead, the CTLH E3 from higher organisms employs a variety of core and auxiliary subunits in addition to GID4 as SRs.

## Regulation by core and higher-order GID/CTLH E3 complex assemblies

### Core GID/CTLH assemblies

**Figure 3:**
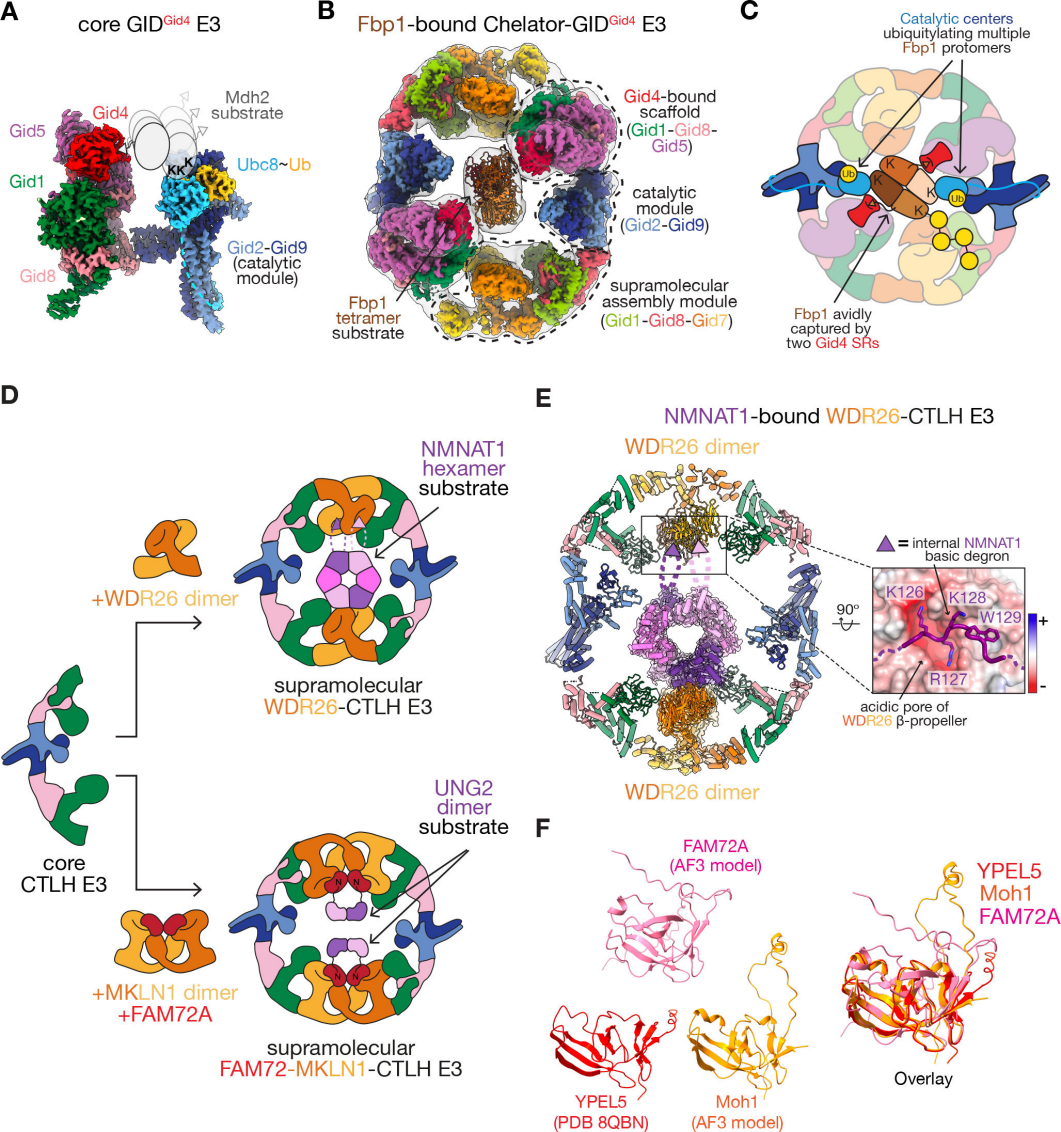
Regulation and substrate specificity by supramolecular GID/CTLH E3 assemblies. (**A**) Composite segmented and color-coded cryo-EM map of the Ubc8~ubiquitin (Ub)-bound core-GID^Gid4^ complex (EMD: 10327 [13] and 17764 [63]). The cartoon of the dimeric Mdh2 substrate was modeled based on the available structural and biochemical data, to represent flexible anchoring of the Mdh2’s Pro/N-degron to Gid4 (gray triangle), enabling its multiple solvent-exposed lysines (K) to access the catalytic center (Ubc8~Ub bound to Gid2-Gid9) for ubiquitylation. **(B)** Composite segmented and color-coded cryo-EM map of Chelator-GID^Gid4^ complex (EMD: 12557, 12560, 12563, 12559) [20] encapsulating the tetrameric Fbp1 substrate (PDB: 7NS5, protomers colored in different shades of brown). The individual functional modules are indicated (dotted lines). **(C)** Cartoon representing the molecular features of the supramolecular Chelator-GID^Gid4^ assembly responsible for efficient oligomeric substrate ubiquitylation: avid binding of Fbp1 tetramer by two opposing Gid4 substrate receptors and ubiquitylation of multiple Fbp1 protomers by two opposing catalytic centers. **(D)** Cartoons of the higher-order WDR26-CTLH and FAM72-MKLN1-CTLH E3 assemblies. Both WDR26 and FAM72-MKLN1 modules serve as substrate receptors for their oligomeric substrates NMNAT1 and UNG2, respectively, positioning them in the hollow center of the oval CTLH E3 complexes. **(E)** Composite structure of the WDR26-CTLH E3 complex bound to its hexameric NMNAT1 substrate. It was generated by docking the structures and AlphaFold3 models of the CTLH modules (PDB: 7NSC, 8PJN) and NMNAT1 crystal structure (PDB: 1KQN [64], protomers colored in different shades of violet and pink) into the cryo-EM map (EMD: 18175) [21]. The high-resolution segmented map of NMNAT1-bound WDR26 dimer is shown as a transparent surface (EMD: 18345) . The close-up depicts the crucial interaction interface between the acidic pore of the WDR26 ß-propeller (shown as the electrostatic potential surface) and NMNAT1’s internal basic degron ‘KRKW’. **(F)** Structure of YPEL5 (PDB: 8QBN) [21], AlphaFold3-predicted FAM72A and yeast Moh1, and their overlay (right) indicating structural similarity.

Cryo-EM structures defined a conserved core catalytic GID/CTLH E3 assembly at high resolution [13,20]. The yeast GID E3 core revealed hallmark E3 ligase features arranged in modules (Figure 3A). First, its catalytic module is formed by an intertwined heterodimer of Gid2 and Gid9, with the coiled-coil domain at the one end and the RING and RING-like domains at the other. The latter activates the dedicated E2 Ubc8~Ubiquitin (Ub) (“~” refers to the high-energy thioester bond between E2’s catalytic Cys and Ub’s C-terminus) for ubiquitin transfer [63]. Second, the catalytic module interacts with a multiprotein scaffold consisting of Gid1, Gid8, and Gid5 (in budding yeast and some higher-eukaryotic CTLH E3 complexes). Gid8 connects the scaffold to the catalytic module through side-by-side interactions between their helical CTLH-CRA^N^ domains, and Gid5 is an adapter for Gid4, Gid10, and Gid11 SRs. Finally, the Gid4’s degron-binding β-barrel domain docks into the crescent-shaped surface composed of Gid5’s helical armadillo repeats [13]. Binding to Gid5 orients Gid4’s substrate-binding site toward the catalytic domains. The core GID E3 resembles a molecular ‘clamp’, whose jaws are formed by Gid4 and the activated Ubc8~Ub. The core GID E3 is tailored to accommodate and ubiquitylate substrates with many ubiquitylation sites tightly tethered by an optimal Pro/N-degron, such as the gluconeogenic substrate Mdh2 (Figure 3A) [13]. Homologous clamp-like architectures have been shown for Gid10-bound yeast core GID E3 complex (GID^Gid10^) and GID4-bound human CTLH E3 (CTLH^GID4^) and are proposed to exist for the Gid11-bound yeast GID E3 complex [13,20]. These E3 architectures suggest a conserved function of Gid5 and its human ortholog ARMC8 in recruiting and orienting SRs toward the catalytic module. Moreover, in yeast, the core GID E3 complex exists in an ‘anticipatory’ state and is activated only upon the recruitment of one of the stress-induced SRs, which allow selective substrate binding and subsequent ubiquitylation (Figure 2D) [11–14,20,47,48,53,54,58].

### Gid7-family subunits assemble ‘Chelator’ E3 architectures

One striking characteristic of the GID/CTLH-family E3 ligases is their ability to form supramolecular assemblies through binding of Gid7-family subunits. This was first identified in yeast, where Gid7, a WD40 repeat β-propeller domain-containing subunit, mediates formation of a giant 1.5-MDa oval-shaped ‘Chelator’-GID^Gid4^ E3 assembly (Figure 3B) [20]. This assembly is named due to its remarkable architecture. The tetrameric gluconeogenic substrate Fbp1 is encapsulated in the hollow center of the E3, reminiscent of an organometallic supramolecular chelate capturing a small ligand through multiple points of contact. The Chelator-GID^Gid4^ E3 assembly consists of 20 GID subunits, comprising two catalytic modules (Gid2-Gid9), two Pro/N-degron SRs (Gid4) bound by two SR-binding scaffolding modules (Gid1-Gid8-Gid5), as well as the two supramolecular assembly modules (Gid1-Gid8-Gid7) (Figure 3B). Within the supramolecular assembly module, the LisH and CRA^C^ domains from two Gid7 protomers promote Gid7 homodimerization [20]. The outward-facing parts of the CTLH and CRA^N^ domains from each Gid7 protomer contact complementary regions from two neighboring copies of Gid1, thereby playing a central part in promoting the oval architecture of the Chelator-GID E3. Fbp1’s degradation relies on its avid binding to two opposing Gid4 SRs and ubiquitylation of its multiple protomers via the two opposing catalytic centers (Figure 3C) [20,63]. This catalytic geometry specifically favors ubiquitin targeting of two Lys pairs located within the allosteric (K32/35) and substrate-binding (K280/281) regions of Fbp1 (Figure 3C). Interestingly, ubiquitylation and degradation of other gluconeogenic enzymes do not require Gid7 [20]. Gid7-independent regulation of Mdh2 may be possible due to its Pro/N-degron binding with higher affinity to Gid4, distinct arrangement of substrate lysines, or other features [13,47].

Intriguingly, Gid7 can mediate formation of even larger assemblies with staggering dimensions of 420 Å x 320 Å x 280 Å, whereby three copies of the Chelator-GID E3 complex are arranged in a ‘cage’-like structure with a large hollow cavity [65]. Although the biological role of this gigantic assembly remains unknown, it is tempting to speculate that the Cage-GID E3 would encapsulate especially large cargoes that would need an extremely large central cavity. Notably, enzymes of metabolic pathways can transiently form functional super-complexes, known as metabolons [66]. Thus, Cage-GID E3 might be designed to target metabolons for ubiquitylation and degradation in pathways yet to be discovered.

### Regulation of substrate specificity by supramolecular CTLH E3 assemblies

Two human Gid7 orthologs, WDR26 and MKLN1, mediate formation of distinct higher-order CTLH E3 assemblies (Figure 3D) [20–22,31,39,50,67,68]. The domain architecture of WDR26 as well as its capacity for homodimerization is analogous to yeast Gid7 [20,67]. In addition to mediating CTLH E3 assemblies, WDR26 is a SR enabling Gid4-independent substrate targeting [21,31,67]. The human WDR26-CTLH E3 assembly targets the transcriptional repressor HBP1 and the metabolic enzyme NMNAT1 for ubiquitin-mediated degradation [21,31,67]. A cryo-EM structure showed NMNAT1 (a homohexameric NAD^+^/NADH biosynthetic enzyme [64,69]) centrally encapsulated within the hollow oval WDR26-CTLH E3, much like the yeast Fbp1-bound Chelator-GID^Gid4^ complex but without Gid4 [20,21] (Figure 3D and E). Instead, the centrally facing WDR26 β-propellers engage two distinct NMNAT1 elements: two protomers within its folded core and basic degron motifs within flexible loops extending from the ordered domains (Figure 3E). The other WDR26-dependent substrate HBP1 shares a related basic motif, suggesting this is a consensus degron for WDR26 [21]. Intriguingly, the basic degron motifs of both NMNAT1 and HBP1 are parts of the nuclear localization sequences, and it is tempting to speculate a functional connection between nuclear localization and protein turnover by the WDR26-CTLH E3 [21]. Mutations in WDR26 are associated with a neurodevelopmental disorder, the Skraban-Deardorff syndrome [70]. Most of the mutations negatively affect the ability of WDR26 to either mediate the supramolecular assembly or engage a substrate, such as HBP1 [71].

The second human Gid7 ortholog, MKLN1, features distinct but related domains, with a β-propeller from the Kelch rather than WD40 family, and an additional discoidin-like domain [72]. Like WDR26, MKLN1 oligomerizes on its own and in the subcomplex with RANBP9-TWA1. However, only the dimeric MKLN1 form assembles within catalytically competent oval higher-order CTLH E3 complexes [20,22,39,50,73–75]. While during mTORC1 inhibition, MKLN1 potentiates Pro/N-degron dependent degradation of HMGCS1 [43–45,47], MKLN1 facilitates degradation of RNA-binding proteins during maternal to zygotic transition in fly embryonic development in the absence of the canonical substrate receptor GID4, which is absent from the fly genome [25,26,75]. Hence, MKLN1 might function as a substrate receptor; however, how it contributes to substrate specificity and ubiquitylation remains unclear. Notably, MKLN1 itself has been suggested to be a substrate of the CTLH E3 complex [25,45,73–75]. Recent work identified FAM72A-MKLN1 as mediating recruitment and ubiquitylation of UNG2, an enzyme involved in the base excision DNA repair pathway, thereby modulating antibody diversification and contributing to neoplasia in a variety of cancer types (Figure 3D) [39,76]. Although the molecular details remain to be resolved, cryo-EM analysis of MKLN1-CTLH E3 complexes both in the absence [20,39] and presence of FAM72A-UNG2 revealed potential to form distinct higher-order oval assemblies, resembling the Chelator-GID and WDR26-CTLH E3 complexes, encapsulating UNG2 dimers in the hollow center [39]. This provides another example that supports the mechanistic concept of supramolecular E3 ligase assemblies tailored for capturing oligomeric proteins [20,21]. Intriguingly, FAM72A is structurally homologous to mammalian YPEL5 and its yeast ortholog Moh1 (Figure 3F) and may define a family of SRs for higher-order GID/CTLH E3 complexes.

### Dynamic variations in CTLH E3 assemblies by paralogous subunits

In addition to Gid7, some of the other mammalian CTLH subunits have multiple functional homologs [16,17]. First, these include the mammalian paralogs RANBP9 (also known as RANBPM) and RANBP10, both orthologous to yeast Gid1. Both of them contain similar functional domains, and hence form analogous scaffolding modules, which are incorporated in the related CTLH E3 assemblies [16,20,22,77]. Expression patterns of RANBP9 and RANBP10 can differ and affect the composition and stoichiometry of RANBP9/10 within CTLH E3 assemblies. For example, RANBP9 is predominantly expressed early and RANBP10 later during erythropoiesis, resulting in differentiation stage-dependent CTLH E3 complexes [30,77]. Second, two orthologs of Gid2 are encoded in the mammalian genome as RMND5A and RMND5B. In contrast to RMND5A [20], RMND5B has not been structurally and biochemically characterized. Recent data suggest a degree of functional redundancy between the RMND5A and RMND5B in targeting UNG2 [39], but unique functions of either paralog remain to be discovered. Finally, the human ortholog of Gid5, ARMC8, is expressed in two isoforms known as ARMC8-α and ARMC8-β [19]. While both isoforms have the ability to assemble into CTLH E3 complexes, only ARMC8-α harbors the GID4 SR-binding domain and thus is able to recruit GID4 [20,65]. Future studies will be required to determine functional differences of these CTLH E3 complexes.

## Regulation by additional modulators of GID/CTLH E3 substrate receptors

Additional cellular factors further modulate substrate receptor activity and specificity. Gid12, an accessory subunit of yeast GID complexes [65,78,79], binds Gid4, buttresses Gid4 to the Gid5 scaffold, and reshapes Gid4’s degron-binding groove [65]. Gid12 also sterically blocks substrates from occupying the center of the oval in Chelator-GID^Gid4^ [65]. While *GID12* deletion has little effect on the levels of yeast gluconeogenic enzymes, overexpression leads to their accumulation under glucose-induced degradation conditions. Future studies will be required to elucidate biological functions of Gid12, and whether these are inhibitory and/or modulating substrate binding.

**Figure 4:**
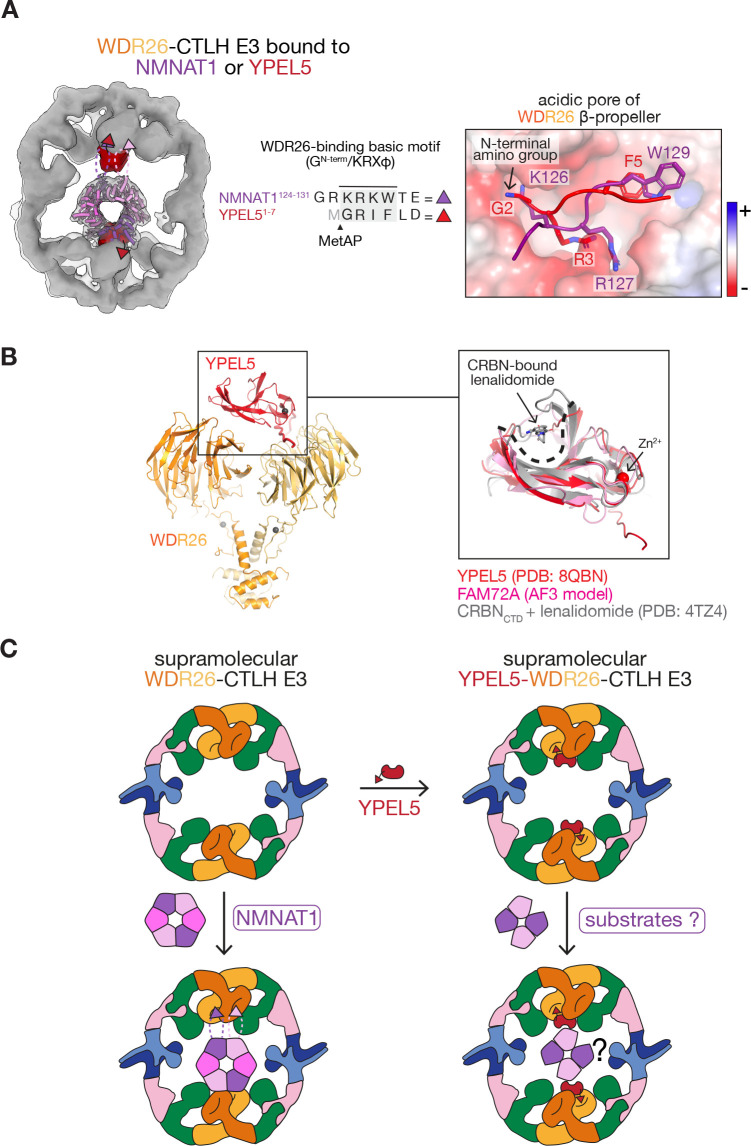
YPEL5 modulates substrate recognition by the WDR26-CTLH E3. (**A**) Left: Overlay of cryo-EM reconstructions of YPEL5-bound (gray solid) and NMNAT1-bound (gray transparent) WDR26-CTLH E3 complexes. Densities of YPEL5 (red) and NMNAT1 (shades of violet) overlap since they are engaged by the common WDR26 β-propeller surface. Right: Notably, the internal basic degron of NMNAT1 (purple) and the N-terminal ‘degron mimic’ of YPEL5 (red) engage the same WDR26 ß-propeller acidic pore. **(B)** Cryo-EM structure of WDR26 dimer bound to YPEL5 (PDB: 8QBN) [21]. Close-up depicts the overlay of YPEL5 and the lenalidomide-bound ‘CULT’ domain of CRBN (gray, PDB: 4TZ4) [80] and AlphaFold3-predicted FAM72A, showcasing their structural similarity. Grey and black spheres indicate Zn^2+^ ions. **(C)** In the absence of YPEL5, the WDR26-CTLH E3 can efficiently engage and ubiquitylate NMNAT1. In contrast, YPEL5 occupies the WDR26 substrate-binding site, inhibiting NMNAT1 targeting. However, due to its well-defined binding pocket and positioning inside the hollow substrate-binding cavity of the CTLH E3, YPEL5 might serve as a receptor for yet unidentified substrates.

Analogous concepts were discovered for the human CTLH E3 subunit YPEL5. One YPEL5 subunit binds both of the β-propellers in a WDR26 dimer [20] and engages similar elements in WDR26 that otherwise bind the substrate NMNAT1 (Figure 4A) [21]. As such, YPEL5’s N-terminus mimics the basic degron and blocks NMNAT1 ubiquitylation *in vitro* and in cells. Accordingly, YPEL5 attenuates cellular NMNAT1 turnover, thereby affecting NMNAT1’s metabolic activity including toward a prodrug [21]. Regulation may involve control of *YPEL5* expression [81]. Indeed, YPEL5 as well as other subunits of the WDR26-CTLH E3 complex are greatly up-regulated during terminal erythroid differentiation, suggesting a differentiation stage-specific function of YPEL5-WDR26 during erythropoiesis [77]. YPEL5 shares a so-called ‘CULT’ domain fold with yeast Moh1, UNG2 receptor FAM72A [39] and also with CRBN [82], which is a SR for the cullin-RING family of modular multiprotein E3s (Figure 4B) [83]. Thus, it seems possible that YPEL5 is not only an inhibitor of some CTLH E3s but could also serve as a SR (Figure 4C).

## Potential for employing CTLH E3s for targeted protein degradation

Targeted protein degradation is an exciting platform in drug discovery, whereby disease-causing proteins are degraded through small-molecules bridging them to E3 ligases [84]. The distinct expression patterns could make various CTLH E3s attractive handles for such therapeutic development. Indeed, GID4 has been the focus of intensive ligand discovery efforts, including NMR-based fragment library screening, DNA-encoded library screening, and affinity selection mass spectrometry screening of a large compound library, and improvements through structure-activity relationships and X-ray crystallography-guided medicinal chemistry [43,44,85]. These studies yielded inhibitors of human GID4 with cellular efficacy [43,44,85] and have enabled discovery of GID4-dependent functions [43]. Notably, GID4-based PROTACs have been recently designed and synthesized to target neo-substrates, such as BRD4, for proteasomal degradation and have been further characterized for their antiproliferative and antitumor growth activity [86]. Furthermore, connecting Pro via chemical linkers to ligands binding ALK fusion and EGFR mutant oncoproteins allowed development of ‘amino acid targeting chimeric molecules’, or aa-TACs, mediating GID4-dependent targeted protein degradation in cells, and showing degradation efficacy in animals too [87].

It is also compelling to consider YPEL5’s structural homology to CRBN (Figure 4B), which is a top E3 currently in development for targeted-protein degradation [80,84,88–90]. YPEL5’s CRBN-like pocket displays distinct molecular features that could in principle be employed for ligand binding [20,21]. Notably, a recent preprint in bioRxiv reports a novel modality—a metabolically-activated charged molecular glue - harnessing this YPEL5 pocket for targeted protein degradation [91].

## Concluding remarks

While the recent advances reviewed here revealed mechanistic concepts that shed light on the versatility of GID/CTLH E3s and their potential to regulate a diverse substrate repertoire, the biological role of these E3 assemblies and their cognate substrates remains largely unknown in higher eukaryotes. Several tempting questions and avenues arise for future investigations. 1) What is the mechanism governing the assembly and/or disassembly of specific CTLH E3 complexes? Identifying cell-type specific conditions as well as cellular triggers that promote expression of GID/CTLH subunits and paralogs will likely uncover mechanisms that favor the preferential formation of GID/CTLH E3 assemblies along with their preferred SRs. 2) Although the canonical SR family Gid4/GID4 has been shown to bind Pro/N- and select non-Pro/N-peptide degrons, only a small number of human GID4 substrates are known so far, and their functional importance remains largely uncharacterized. 3) What can we learn from the conservation and diversity of the GID/CTLH E3s? Intriguingly, certain higher eukaryotes lack orthologs of the key yeast substrate receptor/adaptor subunits Gid4/Gid5, while mammals have expanded repertoire of CTLH E3 subunits. CTLH E3 complexes might have been co-evolved and repurposed along with species-specific diversification of biological processes, allowing alternative targeting mechanisms that yet need to be discovered. Future research will undoubtedly unmask novel aspects of the GID/CTLH E3 family, revealing additional substrates and biological processes they regulate.

**Perspective**

- Ubiquitylation—the reversible, covalent attachment of the small protein ‘ubiquitin’ on other proteins—is one of the most complex and diverse cellular protein modifications that controls seemingly all major biological processes. Specificity of the ubiquitin system is largely determined by E3 ubiquitin ligases that regulate substrate recognition and subsequent ubiquitylation.
- Recent studies of the conserved multiprotein GID/CTLH E3 ubiquitin ligase family revealed new conceptual mechanisms for selective substrate recruitment and ubiquitin targeting. GID/CTLH E3 complex undergoes remarkable structural transformations by incorporating interchangeable and variable subunits, and forming distinct supramolecular assemblies for efficient targeting of a wide repertoire of oligomeric substrates.
- While largely unexplored, future studies are needed to address the biological role of the diverse GID/CTLH E3 assemblies and their substrates in higher eukaryotes. Disease association and the potential for employing CTLH E3 in the field of targeted protein degradation will initiate further investigations for therapeutic applications.

## Abbreviations

CRA: CT11-RanBP9
CTLH: C-terminal to LisH
